# 

**Published:** 1968-10

**Authors:** 


### *Contents*

| | | Page |
|---|---|---|
| VOCATIONAL TRAINING FOR GENERAL PRACTICE .............. | *Michael Lennard* | 23 |
| A NURSE IN GENERAL PRACTICE .............. | *R. W. Morris* | 29 |
| FACTS AND FANCIES IN ORTHOPAEDICS .......................... | *Norman Capener* | 31 |
| A SHORT HISTORY OF ISOLATION ACCOMMODATION IN BRISTOL ........... | *D. W. Wright* | 35 |
| NEWLY APPOINTED CONSULTANTS ............................ | | 38 |
| NOTES & NEWS ...................................................... | | 39 |

